# Effectiveness and safety of brucea javanica oil assisted TACE *versus* TACE in the treatment of liver cancer: a systematic review and meta-analysis of randomized controlled trials

**DOI:** 10.3389/fphar.2024.1337179

**Published:** 2024-06-21

**Authors:** Zhi-Hai Wu, Hai-Feng Zhang, Jun-Yan Li, Yi-Rui Diao, Man-Jing Huang, Dong-Yang Gao, Chang-Hao Liang, Zhi-Qiang Luo

**Affiliations:** ^1^ State Key Laboratory for Quality Ensurance and Sustainable Use of Dao-di Herbs, National Resource Center for Chinese Materia Medica, China Academy of Chinese Medical Sciences, Beijing, China; ^2^ School of Life Sciences, Beijing University of Chinese Medicine, Beijing, China; ^3^ Department of Epidemiology, School of Public Health, Suzhou Medical College of Soochow University, Suzhou, China; ^4^ School of Information Technology, Monash University Malaysia, Subang Jaya, Malaysia; ^5^ Beijing University of Chinese Medicine Third Affiliated Hospital, Beijing, China; ^6^ School of Chinese Materia Medica, Beijing University of Chinese Medicine, Beijing, China; ^7^ Centre for Evidence-Based Chinese Medicine, Beijing University of Chinese Medicine, Beijing, China

**Keywords:** liver cancer, transarterial chemoembolization (TACE), brucea javanica oil (BJO), efficacy, meta-analysis

## Abstract

**Background:** The effectiveness and safety of using Brucea javanica oil (BJO) in combination with Transarterial Chemoembolization (TACE) for liver cancer treatment are subjects of debate. This study aims to assess the comparative effectiveness and safety of BJO-assisted TACE *versus* TACE alone and quantifies the differences between these two treatment methods.

**Methods:** A systematic search was conducted in multiple databases including PubMed, Cochrane, CNKI, and Wanfang, until 1 July 2023. Meta-analysis was conducted, and the results were presented as mean difference (MD), risk ratio (RR), and 95% confidence intervals (CI).

**Results:** The search yielded 11 RCTs, with a combined sample size of 1054 patients. Meta-analysis revealed that BJO-assisted TACE exhibited superior outcomes compared to standalone TACE. Specific data revealed that BJO-assisted TACE improves clinical benefit rate by 22% [RR = 1.22, 95% CI (1.15, 1.30)], increases the number of people with improved quality of life by 32%, resulting in an average score improvement of 9.53 points [RR = 1.32, 95% CI (1.22, 1.43); MD = 9.53, 95% CI (6.95, 12.10)]. Furthermore, AFP improvement rate improved significantly by approximately 134% [RR = 2.34, 95% CI (1.58, 3.46)], accompanied by notable improvements in liver function indicators, with an average reduction of 27.19 U/L in AST [MD = −27.19, 95% CI (−40.36, −14.02)], 20.77 U/L in ALT [MD = −20.77, 95% CI (−39.46, −2.08)], 12.17 μmol/L in TBIL [MD = −12.17, 95% CI (−19.38, −4.97)], and a decrease of 43.72 pg/mL in VEGF [MD = −43.72, 95% CI (−63.29, −24.15)]. Most importantly, there was a 29% reduction in the occurrence of adverse reactions [RR = 0.71, 95% CI (0.60, 0.84)].

**Conclusion:** These findings indicate that BJO-assisted TACE may be considered as a potentially beneficial treatment option for liver cancer patients when compared to standalone TACE. It appears to contribute to improved treatment outcomes, enhanced quality of life, and potentially reduced adverse reactions, suggesting it warrants further investigation as a promising approach for liver cancer treatment.

**Systematic Review Registration:** identifier CRD42023428948

## Introduction

Liver cancer, a prevalent and consequential malignancy worldwide, exhibits a persistent upward trajectory in terms of mortality rates, consistently holding the third position (Wei et al., 2016; Nio et al., 2017; Chen et al., 2020). Liver cancer’s insidious onset often means that symptoms do not appear until the disease has reached intermediate stages, rendering surgical removal less effective (Nio et al., 2017; Zheng et al., 2018). Transarterial chemoembolization (TACE) serves to impede tumor growth and progression as per the Barcelona Clinic Liver Cancer system (Lacaze and Scotté, 2015). After TACE, tissue hypoxia may facilitate the recurrence of liver cancer (Llovet et al., 1999; Chang et al., 2020). To combat this situation, sorafenib is typically used, but it can lead to gastrointestinal discomfort and other adverse reactions (Colagrande et al., 2015; Keating, 2017; Llovet et al., 2021). Currently, we are working diligently to find more suitable drugs to assist in TACE treatment for liver cancer patients.

Traditional Chinese Medicine (TCM) has a long history of successful utilization in the treatment of cancer tumors (So et al., 2019; Xiang et al., 2019). Recent studies have shed light on the remarkable anti-tumor properties of various components derived from specific TCM formulas (Liu et al., 2019; Vitelli Storelli et al., 2019; Zhang et al., 2019). Among these components, Brucea javanica oil (BJO) has exhibited notable abilities in inhibiting tumor cells (Ren et al., 2011; Pan et al., 2018; Tan et al., 2019; Xie et al., 2019; Fan et al., 2020; Li et al., 2021). Meta-analyses have indicated the potential of BJO as a complementary treatment for various cancers, particularly those affecting the digestive system (Jin et al., 2015; Chen et al., 2022). Additionally, numerous studies have reported its clinical efficacy in assisting TACE for liver cancer (J. Chen et al., 2022; Jin et al., 2015). However, there is currently no evidence-based medicine to demonstrate the efficacy and safety of BJO-assisted TACE in the treatment of liver cancer. Therefore, it is imperative to conduct a meta-analysis to evaluate the effectiveness and safety of BJO-assisted TACE *versus* standalone TACE in liver cancer patients. This analysis will provide clinicians with essential insights to find more appropriate, efficient, and secure drug regimens for adjuvant TACE treatment in liver cancer patients.

## Materials and methods

### Review registration

The present systematic review and meta-analysis was conducted in adherence with the PRISMA guidelines and Cochrane Handbook for Systematic Reviews (Moher et al., 2009; Cumpston et al., 2019; Shariati et al., 2023). The study was registered with PROSPERO under the number CRD42023428948. Code and data for this study is publicly available.

### Search strategy

A comprehensive search for relevant studies was conducted in various databases including PubMed, Cochrane Library, Wanfang Data Knowledge Service Platform (Wanfang), and China National Knowledge Infrastructure (CNKI). The search period encompassed the inception of these libraries up to 1 July 2023, with no restrictions on source or language. The following keywords (subject in CNKI and Wanfang) were used for retrieval: 1) ‘liver cancer’; 2) ‘brucea javanica oil’. Subsequently, the two groups were connected using the term ‘AND’. For retrieval, the following keywords (MeSH in PubMed and Cochrane Library) and free words were employed: 1) Liver neoplasm, hepatic neoplasms, hepatic neoplasm, cancer of liver, hepatocellular cancer, hepatocellular cancers, hepatic cancer, hepatic cancers, liver cancer, liver cancers, cancer of the liver; 2) Brucea javanica oil, rhus javanica, java brucea, brucea amarissima. Subsequently, the heading terms and free words within each group were connected using the term ‘or’. The two groups were connected using the term ‘and’. Furthermore, a meticulous review of the relevant literature in the included studies was conducted to ensure the identification of all potential studies.

### Inclusion criteria

Study design: Randomized controlled trials (RCTs); Patient: All patients clinically diagnosed with liver cancer were not limited by age, region, gender, race, or other factors; However, it was ensured that there were no statistically significant differences in age, gender, and liver function grading between the experimental and control groups; Intervention: The control group received TACE as the intervention, while the experimental group received a combination of TACE and BJO; Type of comparison: The experimental group (BJO-assisted TACE) was compared with the control group (TACE alone); Outcomes: Including one of these outcomes: clinical benefit rate (CBR), quality of life (Karnofsky) (Yates et al., 1980; Clancey, 1995), aspartate aminotransferase (AST), alanine aminotransferase (ALT), total bilirubin (TBIL), alpha fetoprotein (AFP) or vascular endothelial growth factor (VEGF).

### Exclusion criteria

All reviews, letters, case reports, conference summaries or records, systematic reviews, scientific and technological achievements and meta-analyses; All animal studies; The outcome data could not be extracted, nor could they be calculated according to the graphs in the article, or the studies obtained by contacting the authors; The experimental group’s intervention included additional types of medicine in addition to BJO and TACE, while the control group’s intervention included additional types of medicine in addition to TACE; No treatment courses recorded.

### Data extraction

Two reviewers (Zhi-Hai Wu and Hai-feng Zhang) extracted independently from the full text of the studies that met the screening criteria. After re-checking with Endnote X9 for Windows (Thomson Reuters, United States) literature management software, the preliminary screening was completed by reading the titles and abstracts, and the full text of potential studies was read to determine whether to include them. If necessary, the authors of the original study can be contacted by email or phone to obtain information of critical importance. All information was independently extracted into a Microsoft Excel spreadsheet, including, if any, country of origin, first author, year of publication, study type, a sample size of patients included, interventions, outcome measures, and outcome data were extracted into a standardized form. Results are checked back-to-back and any discrepancies can be resolved by referring to the original study or consulting a third reviewer (Zhi-qiang Luo).

### Risk of bias assessment

Two independent reviewers evaluated the quality of each enrolled study using the criteria outlined in the Cochrane Handbook for Systematic Reviews of Interventions (version 5.1.0). The assessment covered six key domains: (i) randomization process, (ii) concealment of allocation, (iii) blinding of participants and personnel, (iv) blinding of outcome assessment, (v) handling of incomplete outcome data, and (vi) reporting of outcomes in a non-biased manner. The grading of recommendations assessment, development, and evaluation (GRADE) approach was used to determine the quality of the evidence (Guyatt et al., 2011). Each result is assigned a certainty level (high, moderate, low, or very low) based on the risk of bias, publication bias, indirectness, imprecision, inconsistency, large effect, plausible confounding, and dose–response gradient.

### Statistical analysis

Mean difference (MD) and Risk Ratio (RR) with corresponding 95% confidence intervals (CI) were calculated and reported. Heterogeneity between studies was evaluated using Cochrane Q statistic and I^2^ statistic. I^2^ > 50%, along with a *p*-value <0.10 was treated as an indication for substantial heterogeneity. We used a random effects model in all analyses regardless of heterogeneity measures as evidence has shown more robust effect estimates compared with fixed effect models (Tufanaru et al., 2015). Sensitivity analysis was performed by removing one study at a time to confirm the robustness of outcomes (Higgins et al., 2003). Publication bias was assessed using a funnel plot when the number of included studies exceeded ten.

## Results

### Search results

**FIGURE 1:**
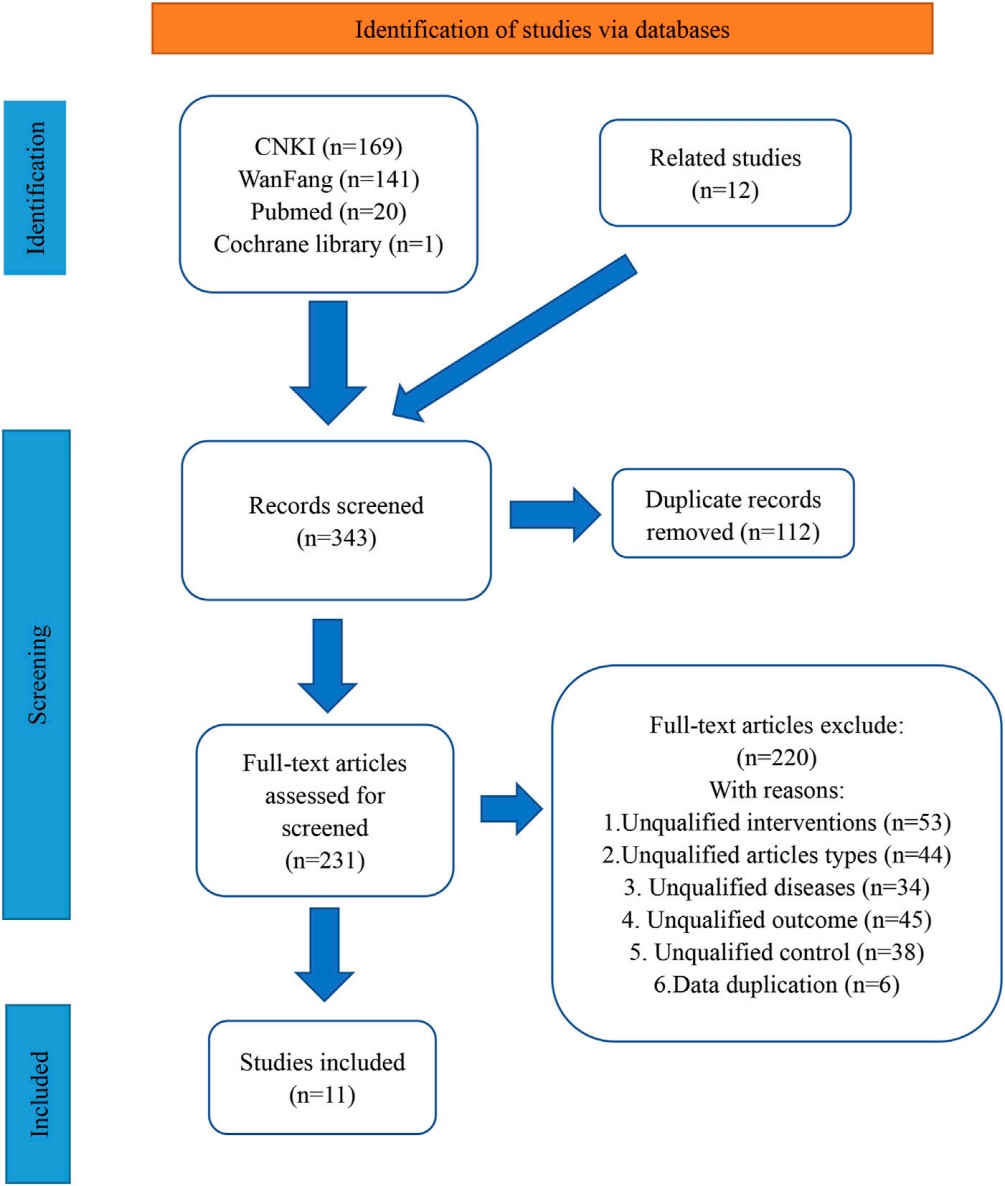
The literature retrieval process Abbreviations: CNKI**,** China National Knowledge Infrastructure. Annotations: Wanfang represents Wanfang Data Knowledge Service Platform

The initial search strategy yielded a total of 343 records. Subsequent removal of duplicates resulted in the identification of 231 unique records. Of these, 220 studies were excluded based on the predetermined inclusion and exclusion criteria. For example, 53 studies were found to be ineligible as they did not meet the criteria of having the control group intervention as TACE alone and the experimental group intervention as BJO-assisted TACE. Following this rigorous selection process, a total of 11 eligible studies were included in the final analysis (Ding and al., 2008; Fu and al., 2016; Huang and al., 2017; Jia and al., 2003; Keyoumu and al., 2017; T. Li and al., 2012; W. Z. Li and Feng, 2006; Y. Li and al., 2016; Lu and al., 2005; Tu and al., 2014; Y. M. Wei and al., 2009). The visual representation of the literature retrieval process was depicted in Figure 1.

### Characteristics of included studies

This study included 11 RCTs conducted within the geographical boundaries of China during the period spanning from 2003 to 2017 (Ding and al., 2008; Fu and al., 2016; Huang and al., 2017; Jia and al., 2003; Keyoumu and al., 2017; T. Li and al., 2012; W. Z. Li and Feng, 2006; Y. Li and al., 2016; Lu and al., 2005; Tu and al., 2014; Y. M. Wei and al., 2009). A total of 1054 patients were included in these trials, with 526 patients assigned to the experimental group and 528 to the control group. The duration of interventions ranged from 6 to 60 days across the various studies. Each study incorporated a minimum of two outcome indicators for comparative analysis. For further details regarding the characteristics of these studies, refer to Table 1.

**TABLE 1:** Basic features of the included studies.

Author	Year	Study design	Country	Sample size		Male/Female		Average age (year)		Intervention		Duration	Outcome
				Exp	Con	Exp	Con	Exp	Con	Exp	Con		
Ding	2008	RCTs	China	32	32	26/6	28/4	No		TACE + BJO	TACE	40d	1,2
Ke	2017	RCT	China	63	63	No		No		TACE + BJO	TACE	15d	1,3.4.5
Li	2012	RCT	China	66	58	50/16	44/14	50.4	51.3	TACE + BJO	TACE	56d	1,2,6
Fu	2016	RCT	China	46	46	No		No		TACE + BJO	TACE	6d	1,2
Wei	2009	RCT	China	45	45	No		No		TACE + BJO	TACE	60d	1,2,6
Jia	2003	RCT	China	34	32	29/5	27/5	No		TACE + BJO	TACE	60d	1,6
Li	2006	RCT	China	39	39	27/12	28/11	No		TACE + BJO	TACE	60d	1,2
Lu	2005	RCT	China	32	30	26/6	25/5	48.2 ± 0.3	47.8 ± 0.6	TACE + BJO	TACE	40d	1,2
Tu	2014	RCT	China	67	63	54/13	52/11	47.3	47.1	TACE + BJO	TACE	30d	1,2
Huang	2017	RCT	China	60	60	36/24	38/22	47.3 ± 7.2	46.8 ± 6.7	TACE + BJO	TACE	60d	1,2,3,4,5
Li	2016	RCT	China	50	50	33/17	31/19	45.4 ± 6.9	48.9 ± 7.1	TACE + BJO	TACE	60d	1,3,4,5

**Abbreviations:** BJO, brucea javanica oil; Con, control group; d, days; Exp, experimental group; RCT, randomized controlled trial; TACE, transarterial chemoembolization.

**Annotations:** 1, Clinical benefit rate (CBR); 2, Quality of life (karnofsky); 3, Aspartate aminotransferase (AST); 4, Alanine aminotransferase (ALT); 5, Total bilirubin (TBIL); 6, Alpha fetoprotein (AFP). No represents that there is no significant difference between experimental group and control group.

### Quality assessment

Table 2 presents the bias risk of 11 studies (Ding and al., 2008; Fu and al., 2016; Huang and al., 2017; Jia and al., 2003; Keyoumu and al., 2017; T. Li and al., 2012; W. Z. Li and Feng, 2006; Y. Li and al., 2016; Lu and al., 2005; Tu and al., 2014; Y. M. Wei and al., 2009). All of these studies employed randomization, but only one study explicitly described the method for concealing the allocation, while the others provided insufficient details regarding blinding procedures. Among the studies included, there were no instances of missing outcome data or selective reporting, resulting in an overall low risk assessment.

**TABLE 2:** Risk of bias assessment.

Source (Author/Year/Country)	Randomization	Allocation concealment	Double-blind	Assessor blinding	Incomplete outcome data addressed	Selective outcome reporting	Other bias
Ding, 2008 China	Low	Unclear	Unclear	Unclear	Low	Low	Low
Li and Feng, 2016 China	Low	Unclear	Unclear	Unclear	Low	Low	Low
Keyoumu, 2017 China	Low	Unclear	Unclear	Unclear	Low	Low	Low
Li 2012 China	Low	Unclear	Unclear	Unclear	Low	Low	Low
Fu 2016 China	Low	Unclear	Unclear	Unclear	Low	Low	Low
Wei 2009 China	Low	Unclear	Unclear	Unclear	Low	Low	Low
Jia 2003 China	Low	Unclear	Unclear	Unclear	Low	Low	Low
Li 2016 China	Low	Unclear	Unclear	Unclear	Low	Low	Low
Lu 2005 China	Low	Unclear	Unclear	Unclear	Low	Low	Low
Tu 2014 China	Low	Unclear	Unclear	Unclear	Low	Low	Low
Huang 2017 China	Low	Low	Unclear	Unclear	Low	Low	Low

### Outcomes

#### Clinical benefit rate (CBR)

**FIGURE 2:**
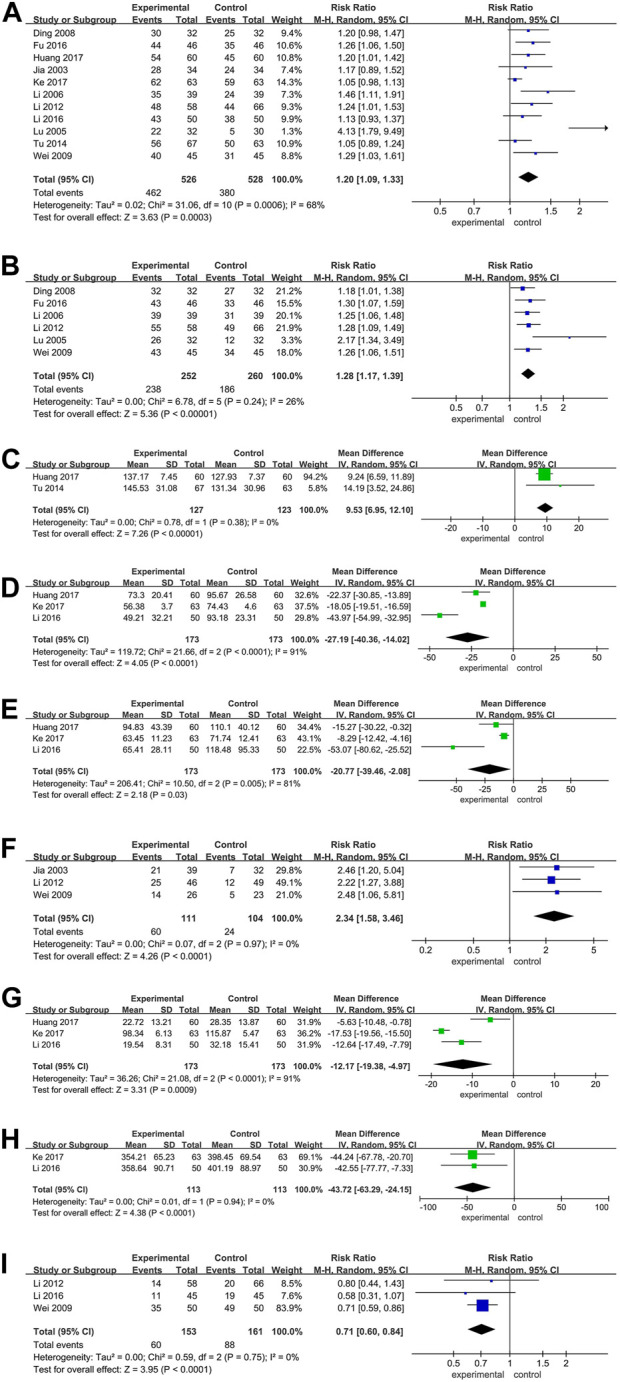
**(A)** Forest plot of the CBR; **(B)** Forest plot of the quality-of-life evaluation (dichotomous data); **(C)** Forest plot of the quality-of-life evaluation (continuous data); **(D)** Forest plot of the AST; **(E)** Forest plot of the ALT; **(F)** Forest plot of the AFP; **(G)** Forest plot of the TBIL; **(H)** Forest plot of the VEGF; **(I)** Forest plot of the adverse reaction. Annotations: In the plot, the 95% confidence interval for each study is represented by the horizontal line and the point estimate is represented by a square. The size of the square is representative of the weight that each study has in the overall effect size estimate. The confidence interval for the overall effect is indicated by a diamond shape at the bottom of the plot. P < 0.05 was considered significant.

All studies compared the clinical benefit rate (CBR) between patients receiving BJO-assisted TACE treatment and those receiving TACE treatment alone (Ding and al., 2008; Fu and al., 2016; Huang and al., 2017; Jia and al., 2003; Keyoumu and al., 2017; T. Li and al., 2012; W. Z. Li and Feng, 2006; Y. Li and al., 2016; Lu and al., 2005; Tu and al., 2014; Y. M. Wei and al., 2009). The meta-analysis results demonstrate a significantly higher CBR in patients who received BJO-assisted TACE treatment compared to those who received TACE treatment alone [RR = 1.22, 95% CI (1.15, 1.30), *p* < 0.001; I^2^ = 68%, *p* < 0.001] (Figure 2A). Sensitivity analysis confirmed the stability of these findings, as there were no significant changes in the results (Supplementary Figure S1). The funnel plot displayed in Supplementary Figure S2 exhibited a cluster of circles in the upper narrow region, suggesting a larger sample size, reduced variance, minimal standard error, and the generation of reliable outcomes.

#### Quality of life

Eight studies compared the quality of Life in patients receiving BJO-assisted TACE treatment and those receiving TACE treatment (Ding and al., 2008; Fu and al., 2016; Huang and al., 2017; T. Li and al., 2012; W. Z. Li and Feng, 2006; Lu and al., 2005; Tu and al., 2014; Y. M. Wei and al., 2009). Among these studies, six used binary data (Ding and al., 2008; Fu and al., 2016; T. Li and al., 2012; W. Z. Li and Feng, 2006; Lu and al., 2005; Y. M. Wei and al., 2009), while the remaining two utilized continuous data (Huang and al., 2017; Tu and al., 2014). Separate analyses were conducted for each type of data. The results of the meta-analysis revealed that, in comparison to patients receiving TACE treatment alone, patients who received TACE treatment with BJO assistance showed a significant increase in both the number of patients experiencing improved quality of life and the average quality of life scores [RR = 1.32, 95% CI (1.22, 1.43), *p* < 0.00001; I^2^ = 26%, *p* = 0.24; MD = 9.53, 95% CI (6.95, 12.10), *p* < 0.00001; I^2^ = 0%, *p* = 0.38] (Figures 2B, C).

#### Aspartate aminotransferase (AST)

Three studies compared patients receiving BJO-assisted TACE treatment with those receiving TACE treatment alone in terms of AST levels (Huang and al., 2017; Keyoumu and al., 2017; Y. Li and al., 2016). The meta-analysis results demonstrated that patients receiving BJO-assisted TACE treatment had significantly lower AST levels than those undergoing TACE treatment alone [MD = −27.19, 95% CI (−40.36, −14.02), *p* < 0.001; I^2^ = 91%, *p* < 0.001] (Figure 2D). Sensitivity analysis did not yield significant alterations in the results (Supplementary Figure S3).

#### Alanine aminotransferase (ALT)

Three studies compared the ALT levels of patients receiving BJO-assisted TACE treatment with those receiving TACE treatment (Huang and al., 2017; Keyoumu and al., 2017; Y. Li and al., 2016). The meta-analysis results showed that patients receiving BJO-assisted TACE treatment had significantly lower AST levels than patients receiving TACE treatment [MD = −20.77, 95% CI (−39.46, −2.08), *p* < 0.05; I^2^ = 81%, *p* < 0.01] (Figure 2E). Sensitivity analysis did not yield significant changes in the results (Supplementary Figure S4).

#### Alpha fetoprotein (AFP)

Three studies compared the levels of AFP in patients receiving BJO-assisted TACE treatment with those receiving TACE treatment (Jia and al., 2003; T. Li and al., 2012; Y. M. Wei and al., 2009). The meta-analysis results indicated that patients receiving BJO-assisted TACE treatment had significantly lower AFP levels compared to those receiving TACE treatment [RR = 2.34, 95% CI (1.58, 3.46), *p* < 0.001; I^2^ = 0%, *p* = 0.97] (Figure 2F).

#### Total bilirubin (TBIL)

Three studies compared the TBIL levels in patients receiving BJO-assisted TACE treatment to those receiving TACE treatment (Huang and al., 2017; Keyoumu and al., 2017; Y. Li and al., 2016). The meta-analysis results revealed a significant decrease in TBIL levels in patients receiving BJO-assisted TACE treatment compared to those receiving TACE treatment [MD = −12.17, 95% CI (−19.38, −4.97), *p* < 0.001; I^2^ = 91%, *p* < 0.001] (Figure 2G). Sensitivity analysis did not produce any significant changes in the results (Supplementary Figure S5).

#### Vascular endothelial growth factor (VEGF)

Two studies compared the levels of TBIL in patients who received BJO-assisted TACE treatment with those who received TACE treatment alone (Keyoumu and al., 2017; Y. Li and al., 2016). The results of a meta-analysis revealed that the VEGF levels in patients who received BJO-assisted TACE treatment were significantly lower than in patients who received TACE treatment alone [MD = −43.72, 95% CI (−63.29, −24.15), *p* < 0.001; I^2^ = 0%, *p* = 0.94] (Figure 2H).

#### Adverse reactions

Two studies compared the adverse reactions in patients who received BJO-assisted TACE treatment and those who received TACE treatment (T. Li and al., 2012; Y. Li and al., 2016). The results of a meta-analysis showed that the adverse reactions in patients who received BJO-assisted TACE treatment were significantly lower than those in patients who received TACE treatment [RR = 0.71, 95% CI (0.60, 0.84), *p* < 0.001; I^2^ = 0%, *p* = 0.75] (Figure 2I).

### Grading the quality of evidence

The GRADE assessment of overall evidence quality ranges from very low to moderate. Specifically, one piece of evidence is of very low quality, three pieces are of low quality, and four pieces are of moderate quality (Table 3).

**TABLE 3:** Quality of evidence of outcomes.

Outcomes	Study design	Risk of bias	Inconsistency	Indirectness	Imprecision	Publication bias	Large effect	Plausible confounding	Dose response gradient	Number of patients		Certainty
										Exp	Con	
CBR	RCTs	Serious^1^	Serious^2^	Not serious	Not serious	Undetected	No	No	No	526	528	Low
Quality of life (a)	RCTs	Serious^1^	Not serious	Not serious	Not serious	Undetected	No	No	No	252	260	Moderate
Quality of life (b)	RCTs	Serious^1^	Not serious	Not serious	Not serious	Undetected	No	No	No	127	123	Moderate
AST	RCTs	Serious^1^	serious^2^	Not serious	Not serious	Undetected	Large	No	No	173	173	Low
ALT	RCTs	Serious^1^	serious^2^	Not serious	Serious^3^	Undetected	Large	No	No	173	173	Low
AFP	RCTs	Serious^1^	Not serious	Not serious	Not serious	Undetected	Large	No	No	111	104	Moderate
TBIL	RCTs	Serious^1^	serious^2^	Not serious	Serious^3^	Undetected	No	No	No	173	173	very Low
VEGF	RCTs	Serious^1^	Not serious	Not serious	Not serious	Undetected	Large	No	No	113	113	Moderate
Adverse reaction	RCTs	Serious^1^	Not serious	Not serious	Not serious	Undetected	No	No	No	153	161	Moderate

**Annotations:** High quality: Further research is very unlikely to change our confidence in the estimate of effect; Moderate quality: Further research is likely to have an important impact on our confidence in the estimate of effect and may change the estimate; Low quality: Further research is very likely to have an important impact on our confidence in the estimate of effect and is likely to change the estimate; Very low quality: The results may vary significantly from the true values, and further research is highly likely to alter the outcomes; ^1^ Inclusion in the study with high or unclear risk of bias (−1); ^2^ Significant heterogeneity (−1); ^3^ Wide confidence intervals (−1).

## Discussion

This study aimed at assessing the effectiveness and safety of BJO-assisted TACE *versus* TACE for liver cancer patients. Our findings highlighted the notable superiority of BJO-assisted TACE over standalone TACE, particularly in terms of improving the clinical benefit rate (CBR), quality of life, alpha-fetoprotein (AFP), aspartate aminotransferase (AST), alanine aminotransferase (ALT), total bilirubin (TBIL), and vascular endothelial growth factor (VEGF) levels in liver cancer patients. Additionally, there was a reduction in the occurrence of adverse reactions associated with TACE treatment (Chang et al., 2020).

This systematic review only considered randomized controlled trials of Grade Ⅰ rating, making its overall findings highly reliable. However, the GRADE assessment revealed a low overall quality of evidence, comprising one very low-quality and three low-quality sources of evidence. This is primarily due to the presence of a high risk of bias in the included studies. To address this issue, we require more relevant high-quality clinical research in the future.

The main components of BJO are Brusatol and Bruceine D. These components primarily exert anticancer effects by inhibiting proliferation and promoting apoptosis. Research has found that Brusatol inhibits the progression of liver cancer by weakening STAT3-driven metastasis in liver cancer cells through altering the levels of epithelial–mesenchymal transition related proteins (Lee et al., 2020). By modulating the PI3K/Akt/mTOR signaling pathway, Brusatol effectively inhibits proliferation, induces apoptosis, and thereby suppresses tumor invasion and migration in liver cancer. This pathway plays a crucial role in signal transduction as well as biological processes such as apoptosis, proliferation, metabolism, and angiogenesis (Ye et al., 2018). The anticancer effect of Brusatol is at least partially mediated by the activation of miRNA-29b expression, inducing p53 and further activating the mitochondrial apoptotic pathway (Wang et al., 2021). Bruceine D is capable of inhibiting the proliferation of liver cancer cells and promoting apoptosis by downregulating the expression of β-catenin and jagged 1 (Cheng et al., 2017). Another study indicates that Bruceine D exerts its anti-liver cancer activity by modulating the expression of miR-95 (Xiao et al., 2014). Therefore, the efficacy of BJO-assisted TACE in the treatment of liver cancer may be achieved through the aforementioned pharmacological mechanisms.

Although BJO shows potential as an adjunct in liver cancer therapy, it is crucial to consider potential limitations and adverse effects. Firstly, the precise dosage and administration regimen of BJO for optimal therapeutic effects are not well-established, which could lead to variability in treatment outcomes and potential toxicity. Additionally, the safety profile of BJO, including its potential interactions with other medications or therapies, requires further investigation. Moreover, while studies have highlighted the anticancer effects of Brusatol and Bruceine D, their specific mechanisms of action and potential side effects in the context of liver cancer treatment need to be elucidated further (Fan et al., 2020). Lastly, the long-term effects of BJO treatment on liver function and overall patient outcomes warrant careful monitoring and investigation.

This study represents the first evaluation of the efficacy and safety of BJO-assisted TACE compared to standalone TACE in the treatment of liver cancer with a quantification of the differences between them. This novel finding provides a more suitable drug option for liver cancer patients, improving treatment outcomes and reducing the risk of adverse reactions associated with TACE. The research expands the treatment options available to clinicians when dealing with liver cancer patients, while also introducing new perspectives in the field of liver cancer treatment and broadening the applications of BJO medications.

### Limitations and prospects

Limitations identified in this study are as follows: Firstly, the absence of clinical guidance pertaining to the optimal dosage and duration of BJO-assisted TACE for the treatment of liver cancer. Secondly, the included studies were predominantly conducted in China, thereby limiting the generalizability of the findings to populations in other geographical regions. Thirdly, although the meta-analysis incorporated randomized clinical trials, which are considered the most dependable study design in medical research, the quality and methodological biases inherent in each individual study may have varied, potentially influencing the pooled results. Finally, the data sources utilized were restricted to Pubmed, Cochrane, CNKI, and Wanfang databases, possibly leading to the exclusion of relevant studies. To comprehensively explore the impact of BJO-assisted TACE on patients with liver cancer and establish comprehensive clinical guidelines for adjunctive medication, future research endeavors should encompass a greater number of high-quality studies while simultaneously endeavoring to minimize the impact of confounding factors, such as clinical variations, methodological disparities, and statistical discrepancies.

## Conclusion

This study suggests potential benefits of BJO-assisted TACE treatment compared to standalone TACE in liver cancer patients, potentially reducing the incidence of adverse reactions associated with TACE. This novel finding introduces promising pharmacological alternatives for TACE combination therapy, offering clinicians additional treatment options for managing liver cancer patients.

## Data Availability

The original contributions presented in the study are included in the article/Supplementary Material, further inquiries can be directed to the corresponding authors.

## Supplementary material

SUPPLEMENTARY FIGURE S1 Sensitivity analysis of the CBR.

SUPPLEMENTARY FIGURE S2 Funnel plot of the CBR. Abbreviations: RR, risk ratio; SE, standard error.

SUPPLEMENTARY FIGURE S3 Sensitivity analysis of the AST.

SUPPLEMENTARY FIGURE S4 Sensitivity analysis of the ALT.

SUPPLEMENTARY FIGURE S5 Sensitivity analysis of the TBIL.

## Abbreviations

AFP, alpha-fetoprotein; ALT, alanine aminotransferase; BJO, brucea javanica oil; CBR, clinical benefit rate; PRISMA, preferred reporting items for systematic reviews and meta-analyses; RCTs, relevant randomized controlled trials; TACE, transarterial chemoembolization; VEGF, vascular endothelial growth factor.
