# Supplementary figures and images for: Effectiveness and safety of brucea javanica oil assisted TACE versus TACE in the treatment of liver cancer: a systematic review and meta-analysis of randomized controlled trials
Source: Front Pharmacol. 2024 Jun 21;15:1337179. doi: 10.3389/fphar.2024.1337179 (PMC11224762; doi:10.3389/fphar.2024.1337179)

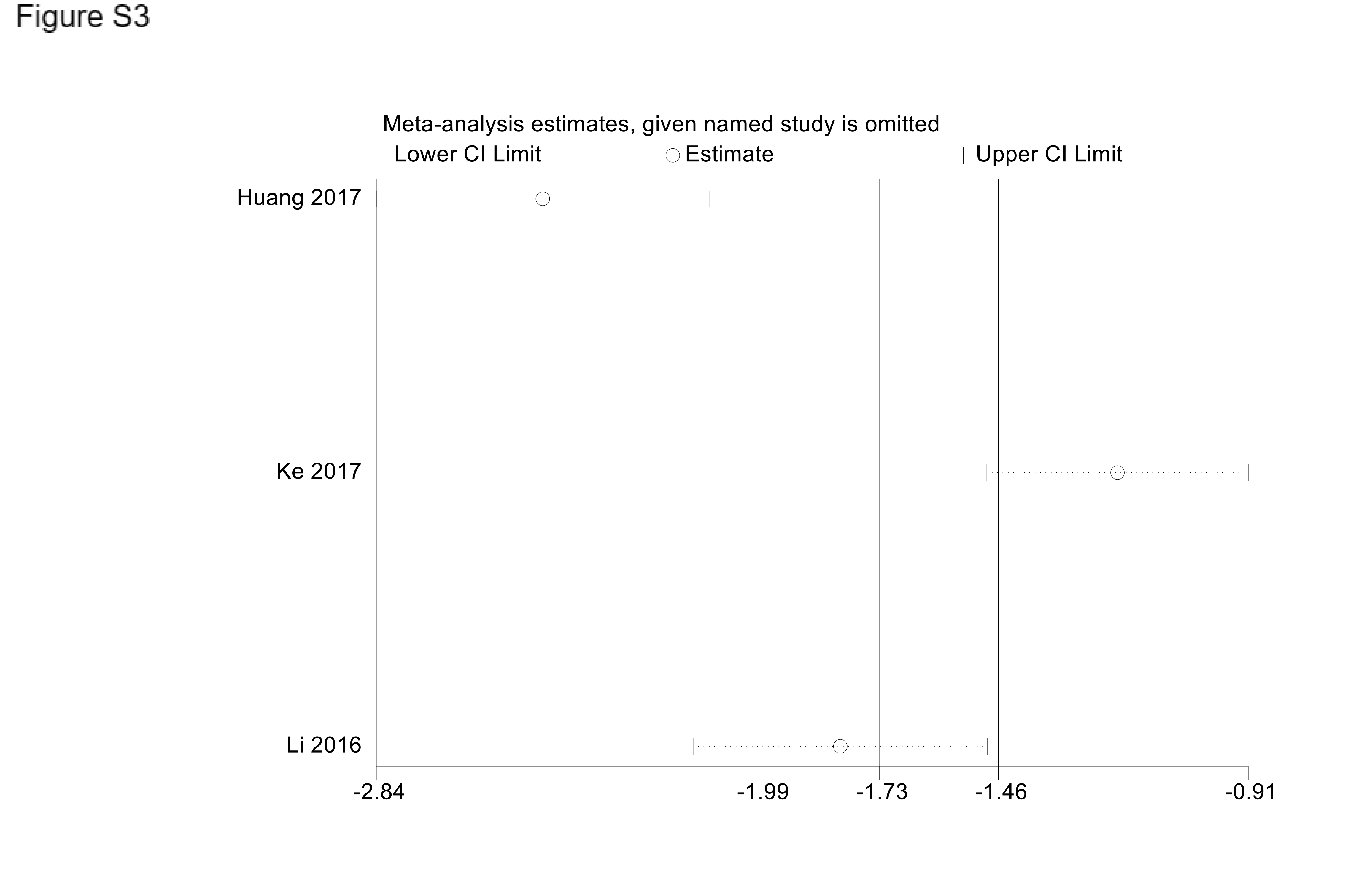

Supplement: Supplementary file 1 [file Image3.tif]

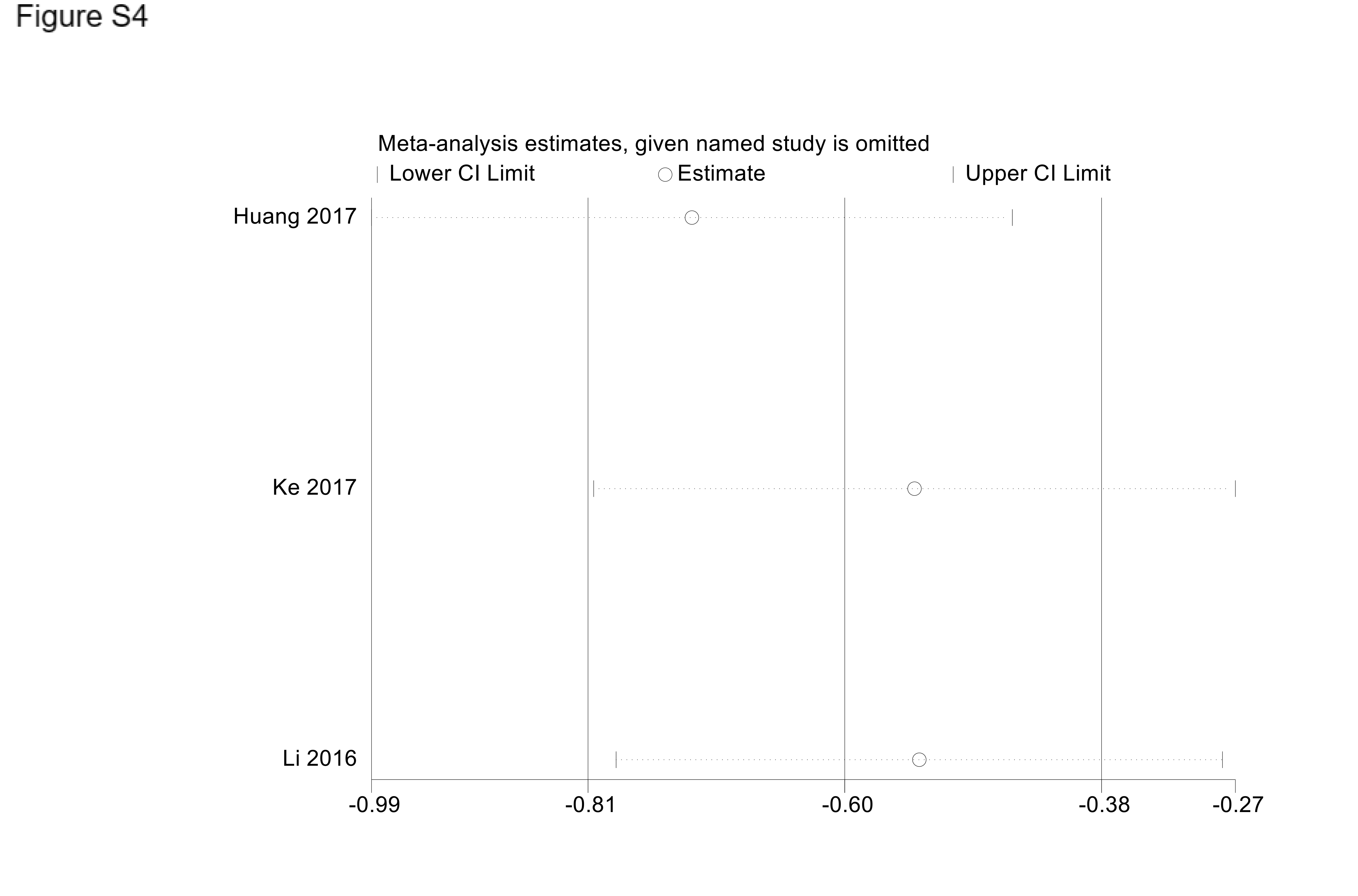

Supplement: Supplementary file 2 [file Image4.tif]

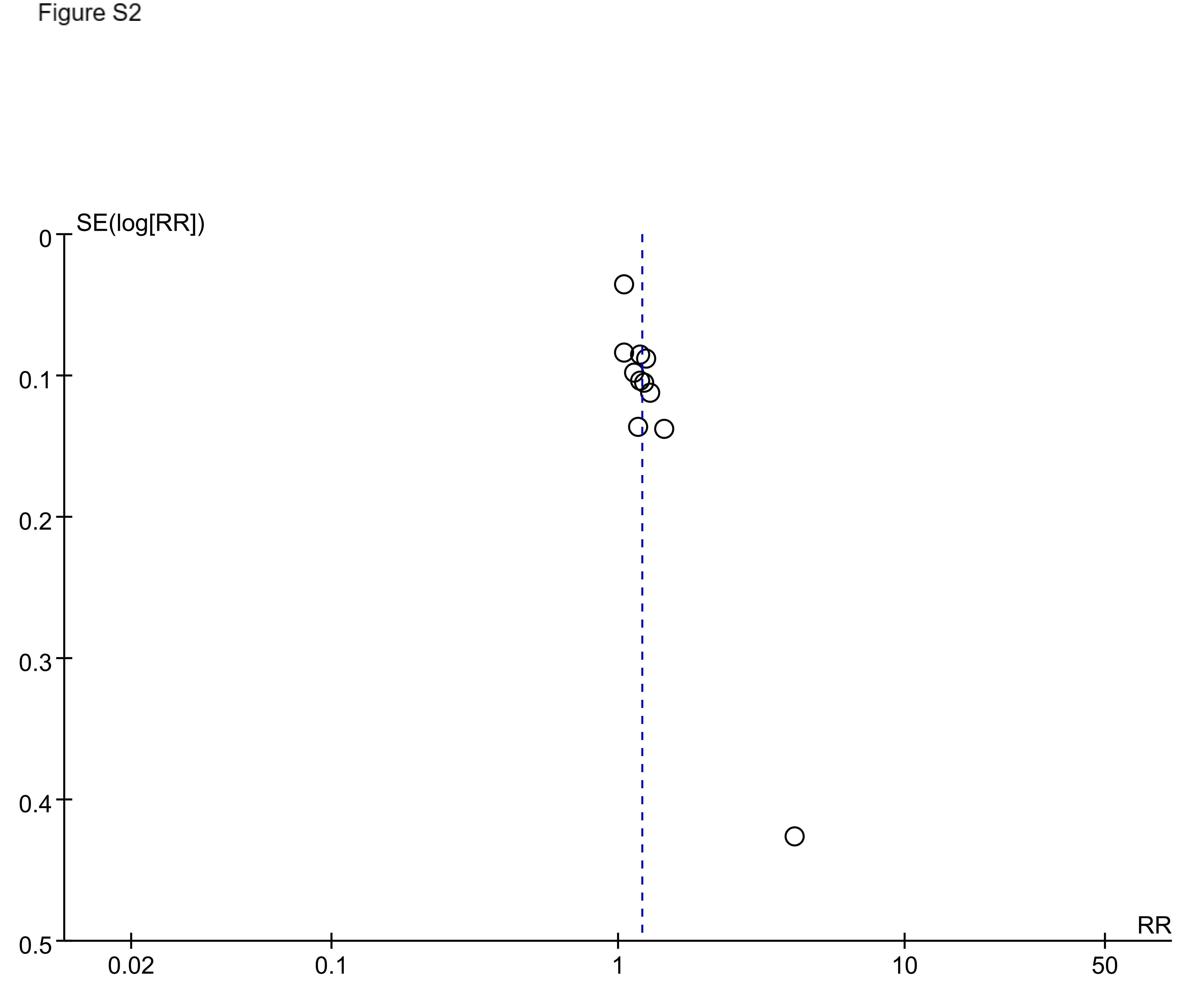

Supplement: Supplementary file 3 [file Image2.tif]

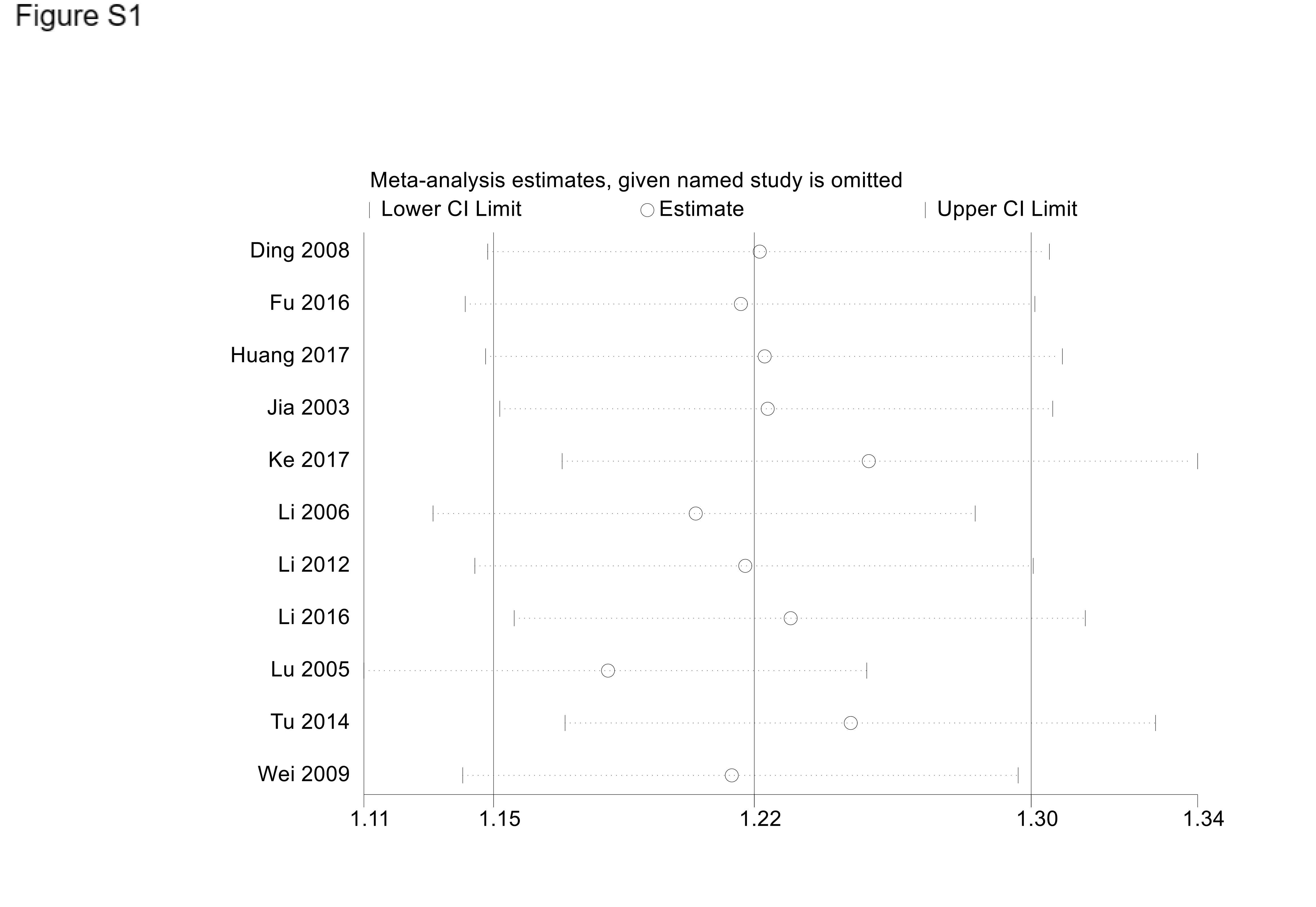

Supplement: Supplementary file 4 [file Image1.tif]

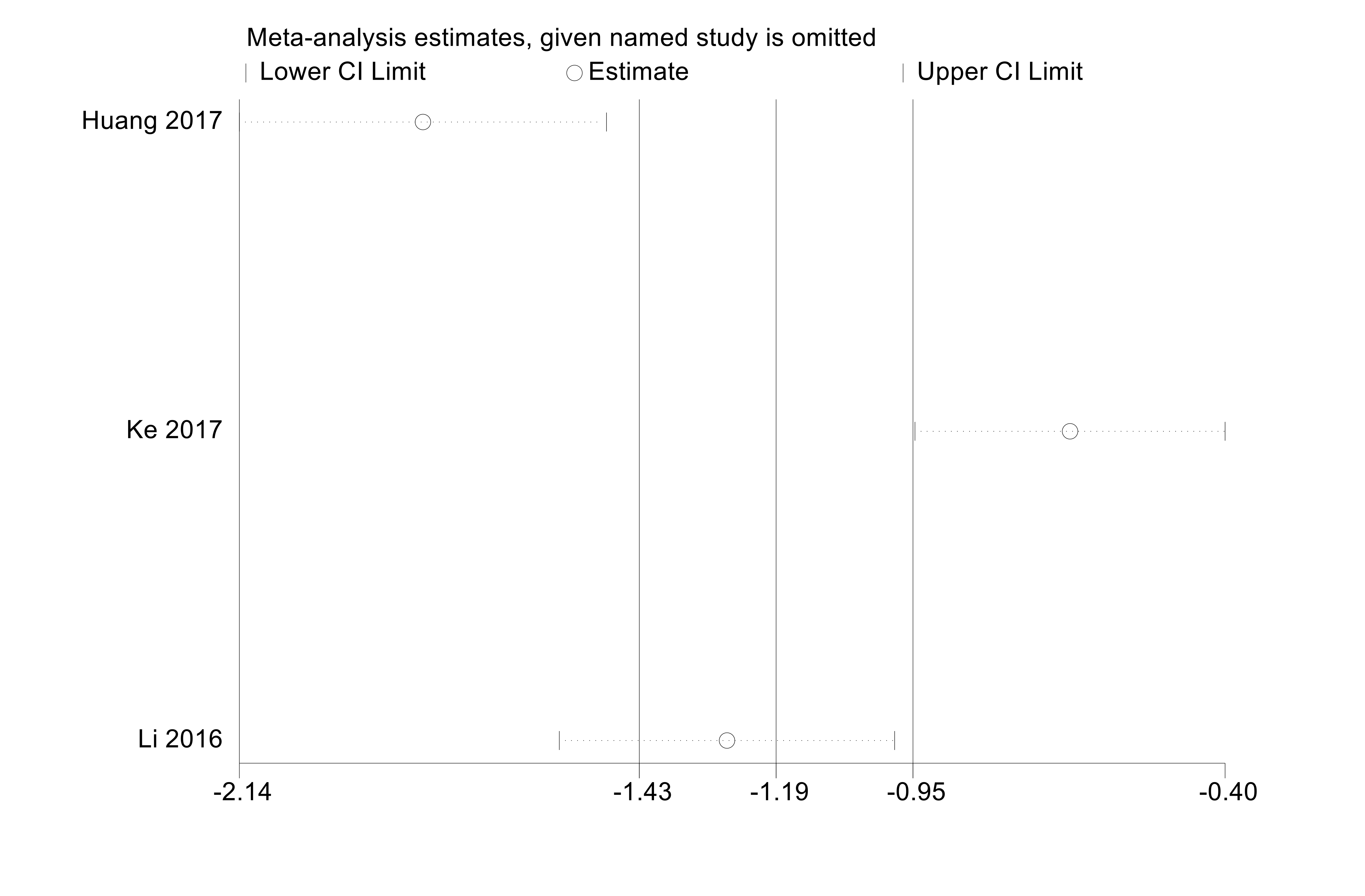

Supplement: Supplementary file 5 [file Image5.tif]
